# Opening the review: perceptions and challenges of Open Science at CSP

**DOI:** 10.1590/0102-311XEN273825

**Published:** 2026-04-10

**Authors:** Luciana Dias de Lima, Claudia Garcia Serpa Osorio-de-Castro, Luciana Correia Alves, Marilia Sá Carvalho

**Affiliations:** 1 Escola Nacional de Saúde Pública Sergio Arouca, Fundação Oswaldo Cruz, Rio de Janeiro, Brasil.; 2 Instituto de Filosofia e Ciências Humanas, Universidade Estadual de Campinas, Campinas, Brasil.; 3 Programa de Computação Científica, Fundação Oswaldo Cruz, Rio de Janeiro, Brasil.

**Keywords:** Peer Review, Scientific and Technical Publications, Open Access, Scientific Publication Ethics, Revisión por Pares, Publicaciones Científicas y Técnicas, Acceso Abierto, Ética en la Publicación Científica

## Abstract

We analyze the perceptions and challenges related to open peer review (OPR) among contributors to *Cadernos de Saúde Pública* (CSP), in the context of Open Science practices. Seeking to understand how authors and reviewers perceive the adoption of this model, a cross-sectional survey was conducted between January and April 2025, with 1,280 respondents among nearly 3,000 Brazilian reviewers from the past three years. The questionnaire, developed on REDCap, consisted of 20 open- and closed-ended questions. Most respondents were female (59.4%), had a PhD degree (70.6%), and ties to public institutions (55.9%) by working in Collective Health research and teaching. As for OPR, while 23.1% were in favor of disclosing the names of authors and reviewers, 24.2% were opposed and 32.7% preferred intermediate answers, revealing caution. Respondents pointed out prior knowledge between authors and reviewers (52.7%) as the main source of discomfort, followed by fears about conflicts of interest and professional constraints. Results indicate that the CSP scientific community recognizes the benefits of OPR for transparency, but also underscore the need for clear guidelines, active editorial mediation, and participant protection. Model acceptance depends on its gradual and contextualized implementation, based on dialogue, training and recognition of review work. In conclusion, OPR can strengthen integrity and trust in science if accompanied by institutional responsibility and sensitivity to the specificities of Collective Health.

## Abstract (es)

Este estudio analiza las percepciones y los retos relacionados con la revisión por pares abierta (*open peer review*; OPR) entre los colaboradores de *Cadernos de Saúde Pública* (CSP), en el contexto de las prácticas de Ciencia Abierta. La investigación buscó comprender cómo los autores y los revisores ven el uso de este modelo. Se trata de una encuesta transversal realizada entre enero y abril de 2025, con 1.280 encuestados entre unos 3.000 colaboradores brasileños en los últimos tres años. El cuestionario, elaborado en REDCap, reunía 20 preguntas abiertas y cerradas. Los encuestados eran predominantemente mujeres (59,4%), con doctorado (70,6%) y profesionales vinculados a instituciones públicas (55,9%), que trabajaban principalmente en investigación y enseñanza en Salud Colectiva. En cuanto a la OPR, el 23,1% se mostró a favor de revelar los nombres de los autores y revisores, mientras que el 24,2% se posicionó en contra; el 32,7% prefirió respuestas intermedias, lo que revela cautela. La principal fuente de incomodidad señalada fue el conocimiento previo entre autores y revisores (52,7%), seguido de los temores sobre conflictos de intereses y tensiones profesionales. Los resultados indican que la comunidad científica de CSP reconoce los beneficios de la OPR para la transparencia, pero también destacan la necesidad de directrices claras, mediación editorial activa y protección de los participantes. La aceptación del modelo depende de su implementación gradual y contextualizada, basada en el diálogo, la formación y el reconocimiento del trabajo de revisión. Se concluye que la OPR puede fortalecer la integridad y la confianza en la ciencia, siempre que vaya acompañada de responsabilidad institucional y sensibilidad hacia las especificidades de la Salud Colectiva.

## Introduction

*Cadernos de Saúde Pública* (CSP) is a journal with over four decades of uninterrupted publication, consolidating itself as one of the most relevant journals in Collective Health research in Latin America. Since 1985, when it published 25 articles in its first volume, CSP has edited over 250 articles per year, in addition to thematic issues on various topics such as aging, technological assessment, child and women’s health, medicines, nutrition, health and environment, and cancer ^1^ ^,^ ^2^. In 2024, the journal celebrated 40 years of continuous publication, reaffirming its role in consolidating the scientific field of Collective Health and its adherence to Open Science practices ^3^.

CSP’s history reveals a continuous process of editorial innovation. In 1998, CSP joined the Scientific Electronic Library Online (SciELO) database, adhering to its principles of interoperability, open access and network collaboration ^4^. This affiliation contributed to strengthening editorial quality, increasing international visibility, and consolidating a scientific culture committed to knowledge as a public good - principles that have been present from the journal’s inception. With support from the Oswaldo Cruz Foundation (FIOCRUZ, acronym in Portuguese) and the Sergio Arouca National School of Public Health (ENSP, acronym in Portuguese), CSP adopts the diamond open access model, ensuring free access and no submission or publication fees, a distinctive characteristic of Latin American journals that are part of the global Open Access Diamond Journals movement ^5^ ^,^ ^6^.

Peer review, a cornerstone of scientific credibility, has been increasingly questioned for its opacity and susceptibility to bias. In response, proposals for open peer review (OPR) have emerged, aiming at greater transparency and traceability in the evaluation process. OPR includes disclosing reviewer and author identities in the article evaluation process, publishing review reports, and, in some cases, making the reviewer-author communication available to the public. International studies show that the OPR model can increase peer review quality and the accountability of reviewers ^7^, although it holds specific challenges in smaller scientific communities ^8^.

OPR practices have been adopted by international institutions. *Plan S* ^9^ and the European Commission’s *Living Guidelines on the Responsible Use of Generative AI in Research* ^10^ reinforce that editorial transparency and the ethical use of emerging technologies are key to strengthening public trust in science. Journals like *Nature Communications* and *eLife* have adopted OPR models that publish authors’ peer reviews and responses to increase transparency, accountability, and collective learning ^11^ ^,^ ^12^.

In Latin America, the discussion about OPR is associated with the commitment of public journals to non-commercial open access. The *Manifiesto sobre la Ciencia como Bien Público Global* [Manifesto on Science as a Global Public Good] ^13^ recommends adopting open models adapted to the region’s cultural and institutional diversity. Aligned with these principles, CSP has advanced in adopting Open Science practices, including the acceptance of preprints (starting in 2020), while maintaining its commitment to editorial integrity and transparency ^3^. Moreover, CSP has been identifying the associate editor responsible for each article since August 2025 and, as of 2026, the names of those reviewers who agree to have their identity revealed ^14^.

Despite these advances, OPR has not been adopted by the journal, partly due to the ethical and social complexities involved. However, the growth of scientific production, the emergence of generative artificial intelligence algorithms, and the increase in article retractions require the permanent reevaluation of scientific review and communication processes. Given this context, understanding how the various academic communities that collaborate with scientific journals perceive and accept the OPR model becomes essential.

Thus, this study evaluated the CSP scientific community’s opinions about OPR. We hope that the present discussion will contribute to improve the journal’s editorial practices and to strengthen scientific integrity and transparency in Collective Health.

## Method

A cross-sectional survey study was conducted with CSP collaborators, using a semistructured online questionnaire to collect information between January and April 2025.

All contributors residing in Brazil (reviewers, authors, and associate editors) affiliated with science and technology institutions in the country, registered in the Article Evaluation and Management System (SAGAS, acronym in Portuguese) hosted on the CSP website, and who had contributed in the last three years, were invited to participate in the study voluntarily and anonymously via email. Of the 3,000 registered contributors, 1,280 participated in the study.

A self-administered questionnaire was developed on the REDCap platform (https://redcapbrasil.com.br/), consisting of 20 closed and open-ended questions organized into modules, with an average response time of approximately 15 minutes and anonymous completion.

The questions covered aspects related to open data and code, and OPR in scientific publications, considering the SciELO criteria ^4^. The questionnaire was divided into modules that collected information on: sociodemographic characteristics, education, career path at CSP, work, open data and code, transparency and OPR. Data were stored on the ENSP server, and all information transmission was carried out via encrypted connection, safeguarding secrecy and confidentiality.

A pilot study was conducted with approximately 20 participants from the Brazilian CSP contributor population, invited via email, to test the instrument and the data collection process.

Data analysis used descriptive statistics, with construction of graphs and tables. Measures of central tendency and dispersion were calculated for the age variable. All data were analyzed on R, version 4.5.1 (http://www.r-project.org), using the *tidyverse* and *ggmosaic* libraries (https://github.com/haleyjeppson/ggmosaic).

The investigation was conducted using CSP’s own resources. The project was approved by the Institutional Review Board of ENSP/FIOCRUZ in March 2024 (CAAE 77523823.9.0000.5240).

## Results

Results reveal diversity of roles in the CSP academic community (603 authors, 220 reviewers, and 349 with both functions). Most contributors had less than five years of affiliation with the journal (53.7%), but a significant percentage reported participation equal to or greater than six years (37.4%), indicating the coexistence of recent and more consolidated trajectories in the editorial field.

From a territorial perspective, most respondents were from the Southeast (45.7%) and South (17.2%) regions, followed by the Northeast (16.6%), Central-West (8%), and North (4.8%). Slightly over half of the participants (57.8%) live in state capitals, whereas 99 respondents (7.7%) did not identify their municipality. This distribution largely reflects the historical concentration of research institutions and graduate programs in Collective Health, while also highlighting the national reach of the CSP collaborator network.

Table 1 summarizes the main characteristics of the respondents. Participants’ sociodemographic profile reveals a higher participation of women (59.4%) and a concentration in the intermediate age groups, especially between 30 and 59 years (70.5%). Mean age was 47.87 years (± 11.97 years) and the median, 46 years. Regarding race/ethnicity, most participants declare themselves White (64.3%) and Mixed-race (22.7%). Albeit small, non-binary people (0.8%) and respondents who chose not to report gender or race/ethnicity were represented, indicating an identity diversity that remains not very expressive in the CSP community. Overall, these data suggest a community composed mostly of middle-aged adult researchers actively involved in the scientific field.

**Table 1:** Sociodemographic characteristics of the sample (n = 1,280).

Characteristics	n	%
Age group (years)		
20-29	73	5.7
30-39	284	22.2
40-49	375	29.3
50-59	243	19.0
60-69	177	13.8
70 and over	41	3.2
Not informed	87	6.8
Gender		
Female	760	59.4
Male	414	32.3
Non-binary person	10	0.8
Prefer not to answer	6	0.5
Not informed	90	7.0
Race/Ethnicity		
White	823	64.3
Mixed-race	291	22.7
Black	54	4.2
Asian	11	0.9
Indigenous	5	0.4
Prefer not to answer	10	0.8
Not informed	86	6.7
Schooling level		
PhD degree	904	70.6
Master’s degree	222	17.3
Specialization/Residence	37	2.9
Undergraduate degree	30	2.3
Not informed	87	6.8
Undergraduate program		
Humanities and Social Sciences	45	3.5
STEM	32	2.5
Health	931	72.7
Not informed	272	21.3
Time since graduation (in years)		
0-9	660	51.6
10-19	326	25.5
20-29	136	10.6
30-39	26	2.0
40 and over	8	0.6
Not informed	124	9.7
Type of employment relationship		
Public servant	716	55.9
CLT	229	17.9
Scholarship recipient	120	9.4
Other	124	9.7
Not informed	91	7.1
Professional activity		
Research/Tertiary education in Collective Health	516	40.3
Research/Tertiary education in other areas	322	25.2
Health management and services	218	17.0
Other	137	10.7
Not informed	87	6.8
Major scientific research areas		
Health Sciences	905	70.7
Applied Social Sciences	85	6.6
Humanities	75	5.9
Biological Science	55	4.3
Exact and Earth Sciences	27	2.1
Engineering	13	1.0
Agricultural Sciences	8	0.6
Linguistics, Language and Literature, and Arts	2	0.2
Not applicable	20	1.6
Not informed	90	7.0

As for academic qualification, most respondents hold a PhD (70.6%) or masters’ degree (17.3%) in Health (72.7%), acting in Health Sciences (70.7%). Although to a lesser extent, areas like Applied Social Sciences, Humanities, and Biological Sciences were also represented, reinforcing the journal’s interdisciplinary nature. Public institutional affiliations (55.9%) and professional activity focused on research and tertiary education in Collective Health (40.3%) predominate, confirming the central role of public institutions in supporting CSP’s editorial activities (Table 1). The significant percentage of respondents who completed their education less than ten years ago (51.6%) suggests a process of academic renewal, with the potential to favor greater openness to editorial innovations.

Perceptions about identity disclosure in the review process reveal a scenario of division and caution (Table 2). Similar proportions of respondents expressed support for and opposition to the full disclosure of authors’ and reviewers’ names (23.1% and 24.2%, respectively), whereas a significant portion preferred intermediate responses like “case by case” or “I do not know” (32.7%; Table 2). Additionally, a smaller fraction stated that they support revealing only the identity of authors (5.6%) or of reviewers (5.7%), indicating that part of the community distinguishes risk and exposure asymmetries between the different roles in the editorial process. This balance between opposing positions suggests that the CSP community recognizes both the potential gains from transparency and the risks of exposure and professional embarrassment, particularly in smaller-scale scientific contexts or those with close interpersonal relationships.

**Table 2:** Respondents’ opinion on open peer review questions (n = 1,280).

Questions	n	%
Are you in favor of disclosing the names of authors, reviewers, or both?		
Neither	310	24.2
Both	296	23.1
Author	72	5.6
Reviewer	73	5.7
I do not know	118	9.2
Case by case	301	23.5
If the identity of editors, reviewers, and authors were fully disclosed, would you refuse to perform a review?		
It depends	461	36.0
No	401	31.3
I do not know	170	13.3
Yes	129	10.1
In which situations below would you feel uncomfortable disclosing your name during the process?		
Author/Reviewer know each other	675	52.7
Know the editor	213	16.6
Topic is controversial	231	18.0
Conflicts of interest	320	25.0
Ethical issues	316	24.7
Assessment indicates refusal	383	29.9

When asked about their willingness to review manuscripts under a fully open system, about one-third stated they would not refuse to do so (31.3%), but most answered “it depends” (36%; Table 2), followed by smaller percentages of uncertainty (“I do not know,” 13.3%) and explicit refusal (10.1%). Such indecisiveness reinforces a cautious and pragmatic attitude towards a model that is still not widely used in the field’s daily practices. Absence of overwhelming rejection, however, suggests a potential opening for change, provided it is accompanied by clear guidelines, active editorial mediation, and measures to protect the integrity of those involved.

Discomfort-generating situations confirm that the main source of apprehension lies in the possibility that authors and reviewers may know each other (52.7%), followed by concerns related to conflicts of interest, evaluations that may result in manuscript rejection, negative assessments, and ethical dilemmas. These results show that resistance to open review is not limited to individual fears, but involves structural perceptions about fairness, impartiality, and preserving critical autonomy in the editorial process. The smaller percentage of those who reported feeling no discomfort at all (15.9%) reinforces the sensitive, relational, and contextual nature of OPR.

**Figure 1:**
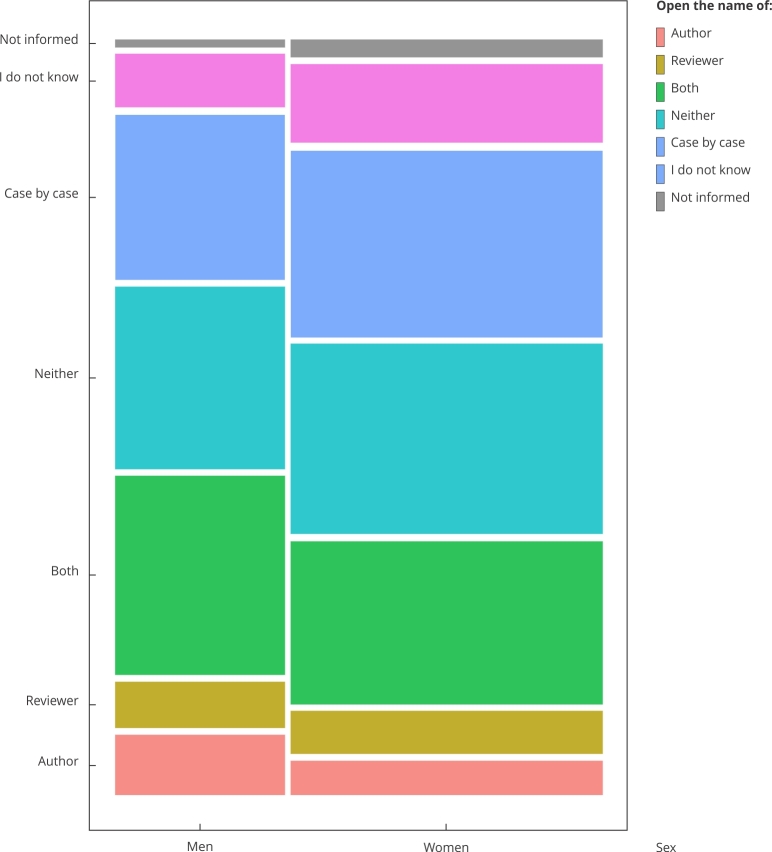
Opinion regarding data openness.

Stratified analysis by self-reported gender reveals statistically significant differences in the distribution of opinions on identity disclosure in the review process (chi-squared test, p < 0.05; Figure 1).

 Women are proportionally more concentrated in the intermediate categories (“case by case” and “I do not know”), whereas men show a higher relative frequency in the extreme positions, both in favor of and against. These findings suggest greater caution among women regarding the adoption of fully open models, especially concerning the identification of authors.

## Discussion

Our findings confirm that peer review is undergoing a historical inflection point, marked by tensions between the need to increase transparency, strengthen integrity in scientific publication, and editorial sustainability, and the structural limitations of the traditional evaluation model. As widely discussed in the literature, peer review, conceived in the 18th century as a practice restricted to scientific elites, has become progressively inadequate before the global expansion of science, the exponential growth of submissions, and the intensification of demands on an increasingly limited number of experienced reviewers ^15^ ^,^ ^16^.

Results show that these contradictions are clearly perceived by the CSP scientific community. The high academic qualifications of the respondents, mostly PhD holders, working in public institutions and with established experience in Collective Health, lend strength to the expressed opinions. As a group involved in the dynamics of production, evaluation, and circulation of scientific knowledge, the predominant intermediate and cautious positions regarding OPR become particularly relevant.

Absence of polarization between positions clearly in favor of or against disclosing author/reviewer identities reinforces previous findings in the national literature. Studies with editors of Brazilian scientific journals indicate that adoption of OPR practices is conditioned by the editorial context, disciplinary culture, and characteristics of the scientific communities, with a recurring defense of gradual and diverse implementation models ^17^ ^,^ ^18^. The significant proportion of “case by case” or “I do not know” responses suggests that the CSP community shares this pragmatic and reflective perspective.

Respondents’ partial reluctance to revise manuscripts in a fully open system also resonates with both international and national literature. Although only a minority explicitly state that they would refuse to review under OPR, predominance of the “it depends” response indicates that adherence to the model will not occur automatically. This result aligns with criticisms of the so-called “peer review pipeline”, characterized by reviewer overload, low academic value of this activity, and the absence of adequate institutional incentives ^15^. In this scenario, OPR, if implemented without safeguards, could exacerbate the shortage of reviewers, especially in areas with relatively small and highly interconnected scientific communities.

The discomfort-generating situations reported by respondents further deepens this analysis. Their main source of concern - that authors and reviewers may know each other’s identities - highlights that the debate on OPR is not limited to formal transparency, but involves ethical, relational, and structural dimensions of science. Studies highlight that, while open review can increase accountability and the argumentative quality of opinions, it can also inhibit more forceful criticism, especially when power imbalances, hierarchical relationships, or prior institutional ties exist ^7^ ^,^ ^19^.

Differences by self-reported gender reveal that gender bias persists even in OPRs ^20^ ^,^ ^21^. The predominance of women in intermediate positions reflects caution in the face of structural asymmetries and reputational risks ^22^, as female researchers face greater exposure to interpersonal judgments and unequal power relations ^23^.

In Brazil, these concerns are particularly relevant. Research with editors of journals indexed in the Directory of Open Access Journals (DOAJ; https://doaj.org/) indicates that OPR tends to be perceived as ethically desirable but operationally complex, requiring careful editorial mediation, clearly defined roles, and strengthening the dialogical dimension of the evaluation process ^17^ ^,^ ^24^. Concrete experiences reported by national journals, such as the journal *Ensaio Pesquisa em Educação em Ciências*, evince that OPR can be successful when accompanied by transparent communication, training of reviewers and explicit recognition of the review work ^25^.

Internationally, the literature reinforces that there is no single model for OPR. Different arrangements - such as publishing reviews without mandatory reviewer identification, optional disclosure of identities, or publicizing editorial dialogues after the final decision - have been adopted with varying results ^16^ ^,^ ^19^. These intermediate models are often cited as viable alternatives for increasing transparency without compromising the critical autonomy of reviewers, especially in sensitive or highly relational areas.

In the Latin American context, and particularly within the framework of diamond open access journals like CSP, the discussion about OPR takes on specific dimensions. Unlike large commercial journals, CSP operates under the principles of science as a public good, supported by public institutions and committed to equity, inclusion, and social responsibility. In this regard, OPR should be understood not only as a technical innovation, but as an ethical and political practice, aligned with Open Science principles and the recommendations of regional and global non-commercial open access initiatives ^13^.

Finally, our study results indicate that the eventual adoption of OPR at CSP should be preceded by extensive dialogue with the community. Continuous educational programs, clearly defined editorial criteria, and mechanisms to protect reviewers, especially early-career researchers and those belonging to historically underrepresented groups, should be considered. To fulfill its promise of greater integrity and public trust in science, open review must be accompanied by institutional accountability, recognition of editorial work, and effective appreciation of peer review as a core activity in scientific practice.

One limitation of the study was the voluntary participation, which may have introduced bias in population characterization, thus restricting the generalizability of the results. Moreover, the cross-sectional design captures perceptions at a specific moment in the debate on OPR, a topic that is rapidly evolving, indicating the need for future studies that allow us to track the unfolding of the issue in the Brazilian academic community.

Our study contributes to the contemporary debate on the subject by showing that the future of peer review does not lie in simply replacing one model with another, but in the collective construction of more transparent, fair, and sustainable arrangements that are sensitive to the specificities of Collective Health and consistent with open science principles.

## Data Availability

The databases used in the study, including extraction codes, analyses, and results, are available in the repository: https://github.com/haleyjeppson/ggmosaic..
