# Supplementary material for: Opening the review: perceptions and challenges of Open Science at CSP
Source: Cad Saude Publica. 2026 Apr 10;42:e00273825. doi: 10.1590/0102-311XEN273825 (PMC13078780; doi:10.1590/0102-311XEN273825)
Supplement: Translation

# Translation: Abrindo a revisão: percepções e desafios da Ciência Aberta em CSP

**DOI:** 10.1590/0102-311XPT273825

**Authors:** Luciana Dias de Lima, Claudia Garcia Serpa Osorio-de-Castro, Luciana Correia Alves, Marilia Sá Carvalho

**Affiliations:** 1 Escola Nacional de Saúde Pública Sergio Arouca, Fundação Oswaldo Cruz, Rio de Janeiro, Brasil.; 2 Instituto de Filosofia e Ciências Humanas, Universidade Estadual de Campinas, Campinas, Brasil.; 3 Programa de Computação Científica, Fundação Oswaldo Cruz, Rio de Janeiro, Brasil.

**Keywords:** Revisão por Pares, Publicações Científicas e Técnicas, Acesso Aberto, Ética na Publicação Científica

## Abstract

Este estudo analisa as percepções e os desafios relacionados à revisão por pares aberta (*open peer review*; OPR) entre colaboradores de *Cadernos de Saúde Pública* (CSP), no contexto das práticas de Ciência Aberta. A pesquisa buscou compreender como autores e pareceristas percebem a adoção desse modelo. Trata-se de um inquérito transversal realizado entre janeiro e abril de 2025, com 1.280 respondentes entre cerca de 3 mil colaboradores brasileiros nos últimos três anos. O questionário, elaborado no REDCap, reuniu 20 questões abertas e fechadas. Os respondentes eram predominantemente mulheres (59,4%), com doutorado (70,6%) e profissionais vinculados a instituições públicas (55,9%), com atuação majoritária em pesquisa e ensino em Saúde Coletiva. Quanto à OPR, 23,1% foram favoráveis à abertura de nomes de autores e pareceristas, enquanto 24,2% se posicionaram contrariamente; 32,7% preferiram respostas intermediárias, revelando cautela. A principal fonte de desconforto apontada foi o conhecimento prévio entre autores e revisores (52,7%), seguida de receios quanto a conflitos de interesse e constrangimentos profissionais. Os resultados indicam que a comunidade científica de CSP reconhece os benefícios da OPR para a transparência, mas também ressaltam a necessidade de diretrizes claras, mediação editorial ativa e proteção aos participantes. A aceitação do modelo depende de sua implementação gradual e contextualizada, com base em diálogo, formação e reconhecimento do trabalho de revisão. Conclui-se que a OPR pode fortalecer a integridade e a confiança na ciência, desde que acompanhada de responsabilidade institucional e sensibilidade às especificidades da Saúde Coletiva.

## Introdução

*Cadernos de Saúde Pública* (CSP) é um periódico com mais de quatro décadas de publicação ininterrupta, consolidando-se como uma das revistas mais relevantes da área da Saúde Coletiva na América Latina. Desde 1985, quando publicou 25 artigos em seu primeiro volume, CSP passou a editar mais de 250 artigos por ano, além de fascículos temáticos sobre temas variados, como envelhecimento, avaliação tecnológica, saúde da criança e da mulher, medicamentos, nutrição, saúde e ambiente e câncer ^1^ ^,^ ^2^. Em 2024, o periódico comemorou 40 anos de trajetória contínua, reafirmando seu papel na consolidação do campo científico da Saúde Coletiva e sua adesão às práticas da Ciência Aberta ^3^.

A história da revista evidencia um processo contínuo de inovação editorial. A partir de 1998, CSP ingressou na base SciELO, aderindo aos seus princípios de interoperabilidade, acesso aberto e colaboração em rede ^4^. Essa adesão contribuiu para o fortalecimento da qualidade editorial, o aumento da visibilidade internacional e a consolidação de uma cultura científica comprometida com o conhecimento como bem público, princípios presentes desde a origem do periódico. Com apoio da Fundação Oswaldo Cruz (FIOCRUZ) e da Escola Nacional de Saúde Pública Sergio Arouca (ENSP), CSP adota o modelo de acesso aberto diamante (*diamond open access*), assegurando acesso gratuito e ausência de taxas de submissão e publicação, uma característica distintiva dos periódicos latino-americanos que integram o movimento global do Open Access Diamond Journals ^5^ ^,^ ^6^.

A revisão por pares, pilar da credibilidade científica, tem sido crescentemente questionada por sua opacidade e suscetibilidade a vieses. Em resposta, surgem propostas de revisão por pares aberta (*open peer review* - OPR), que visam maior transparência e rastreabilidade no processo de avaliação. Essa modalidade pode incluir a revelação das identidades de autores e revisores no processo de avaliação do artigo, a publicação dos pareceres e, em alguns casos, a abertura pública da discussão. Estudos internacionais mostram que a OPR pode aumentar a qualidade dos pareceres e a responsabilidade dos revisores ^7^, ainda que apresente desafios específicos em comunidades científicas menores ^8^.

A adoção de práticas de avaliação aberta vem sendo promovida por instituições internacionais. O *Plan S* [Plano S] ^9^ e as *Living Guidelines on the Responsible Use of Generative AI in Research* [Diretrizes Vivas sobre o Uso Responsável da IA ​​Generativa em Pesquisa] da Comissão Europeia ^10^ reforçam que a transparência editorial e o uso ético de tecnologias emergentes são fundamentais para fortalecer a confiança pública na ciência. Revistas como *Nature Communications* e *eLife* adotaram modelos de OPR, que publicam os pareceres e as respostas dos autores para aumentar a transparência, a responsabilidade e o aprendizado coletivo ^11^ ^,^ ^12^.

Na América Latina, a discussão sobre OPR está associada ao compromisso das revistas públicas com o acesso aberto não comercial. O *Manifiesto sobre la Ciencia como Bien Público Global* [Manifesto sobre a Ciência como um Bem Público Global] ^13^ recomenda a adoção de modelos abertos adaptados à diversidade cultural e institucional da região. CSP, alinhada a esses princípios, avançou na adoção de práticas de Ciência Aberta, incluindo a aceitação de *preprints* (a partir de 2020), mantendo seu compromisso com a integridade e a transparência editorial ^3^. Além disso, desde agosto de 2025, a revista passou a identificar o editor associado responsável por cada artigo e, a partir de 2026, os nomes dos revisores que aceitarem ter a sua identidade revelada ^14^.

Apesar desses avanços, a OPR não foi adotada pela revista, em parte devido às complexidades éticas e sociais envolvidas. No entanto, o crescimento da produção científica, a emergência de algoritmos de inteligência artificial generativa e o aumento de retratações de artigos exigem a reavaliação permanente dos processos de revisão e comunicação científica. Nesse contexto, torna-se essencial compreender como as diversas comunidades acadêmicas, que colaboram com as revistas científicas, percebem e aceitam o modelo de OPR.

O artigo tem como objetivo avaliar a opinião da comunidade científica vinculada à CSP sobre a OPR. Espera-se, com isso, contribuir para o aperfeiçoamento das práticas editoriais do periódico e para o fortalecimento da integridade e da transparência científica no campo da Saúde Coletiva.

## Método

Realizou-se um estudo transversal do tipo inquérito com os colaboradores de CSP, utilizando-se um questionário online semiestruturado para a coleta de informações no período de janeiro a abril de 2025.

Todos os colaboradores que residem no Brasil (revisores, autores e editores associados) inseridos em instituições de ciência e tecnologia do país, constantes no cadastro do Sistema de Avaliação e Gerenciamento de Artigos (SAGAS), hospedado no site de CSP, e que tinham contribuído nos últimos três anos, foram convidados a participar do estudo de forma voluntária e anônima, por e-mail. De um total de 3 mil colaboradores cadastrados, 1.280 participaram.

O formulário de autopreenchimento foi elaborado na plataforma REDCap (https://redcapbrasil.com.br/), composto por 20 questões fechadas e abertas, organizadas em módulos, com tempo médio de resposta de aproximadamente 15 minutos, e preenchido de forma anônima.

As questões contemplavam aspectos relacionados aos dados e códigos abertos e à OPR em publicações científicas, considerando os critérios do SciELO ^4^. O questionário foi dividido em módulos que coletaram informações sobre: características sociodemográficas, formação, trajetória em CSP, trabalho, dados e códigos abertos e transparência e abertura de avaliação por pares. Os dados coletados foram armazenados em servidor da ENSP e toda a transmissão de informação realizada por conexão criptografada, resguardando sigilo e confidencialidade.

Um estudo piloto foi conduzido com aproximadamente 20 participantes da população de colaboradores brasileiros de CSP, convidados por e-mail, com o objetivo de testar o instrumento e o processo de coleta de dados.

A análise dos dados foi feita por meio de estatística descritiva, com construção de gráficos e tabelas. Medidas de tendência central e de dispersão foram calculadas para a variável idade. Os dados foram analisados no software R, versão 4.5.1 (http://www.r-project.org), por meio das bibliotecas *tidyverse* e *ggmosaic* (https://github.com/haleyjeppson/ggmosaic).

O inquérito foi feito com recursos próprios de CSP. O projeto foi aprovado pelo Comitê de Ética em Pesquisa (CEP) da ENSP/FIOCRUZ, em março de 2024 (CAAE 77523823.9.0000.5240).

## Resultados

O estudo revela a diversidade de atribuições da comunidade acadêmica vinculada à CSP (603 autores, 220 pareceristas e 349 com ambas as funções). Observou-se predominância de colaboradores com vínculo inferior a cinco anos com o periódico (53,7%), embora uma parcela expressiva relate participação igual ou superior a seis anos (37,4%), indicando a coexistência de trajetórias recentes e mais consolidadas no âmbito editorial.

Do ponto de vista territorial, verificou-se maior concentração de respondentes nas regiões Sudeste (45,7%) e Sul (17,2%), seguidas pelo Nordeste (16,6%), Centro-oeste (8%) e Norte (4,8%). Pouco mais da metade dos participantes (57,8%) moravam nas capitais dos estados, sendo que um total de 99 informantes (7,7%) não identificou sua região. Essa distribuição regional reflete, em grande medida, a concentração histórica de instituições de pesquisa e programas de pós-graduação na área da Saúde Coletiva, ao mesmo tempo em que evidencia a abrangência nacional da rede de colaboradores de CSP.

Na Tabela 1, é possível observar as principais características dos respondentes. O perfil sociodemográfico dos participantes revela maior participação de mulheres (59,4%) e concentração nas faixas etárias intermediárias, especialmente entre 30 e 59 anos (70,5%). A média de idade foi de 47,87 anos (± 11,97 anos). A mediana foi de 46 anos. Em relação à raça/cor da pele, predominam pessoas que se autodeclaram brancas (64,3%) e pardas (22,7%). Ainda que em proporção reduzida, registrou-se a presença de pessoas não binárias (0,8%) e de respondentes que optaram por não informar gênero ou raça/cor da pele, indicando a existência de diversidade identitária ainda pouco expressiva na comunidade de CSP. Em conjunto, esses dados sugerem uma comunidade composta por pesquisadores majoritariamente adultos de meia-idade, com inserção ativa no campo científico.

**Tabela 1:** Características sociodemográficas da amostra (n = 1.280).

Características	n	%
Faixa etária (anos)		
20-29	73	5,7
30-39	284	22,2
40-49	375	29,3
50-59	243	19,0
60-69	177	13,8
70 e mais	41	3,2
Sem informação	87	6,8
Gênero		
Mulher	760	59,4
Homem	414	32,3
Pessoa não binária	10	0,8
Prefiro não responder	6	0,5
Sem informação	90	7,0
Raça/Cor da pele		
Branca	823	64,3
Parda	291	22,7
Preta	54	4,2
Amarela	11	0,9
Indígena	5	0,4
Prefiro não responder	10	0,8
Sem informação	86	6,7
Nível de escolaridade		
Doutorado	904	70,6
Mestrado	222	17,3
Especialização/Residência	37	2,9
Graduação	30	2,3
Sem informação	87	6,8
Curso de graduação		
Sociais e Humanas	45	3,5
STEM	32	2,5
Saúde	931	72,7
Sem informação	272	21,3
Tempo de conclusão do curso (em anos)		
0-9	660	51,6
10-19	326	25,5
20-29	136	10,6
30-39	26	2,0
40 e mais	8	0,6
Sem informação	124	9,7
Tipo de vínculo		
Servidor público	716	55,9
CLT	229	17,9
Bolsista	120	9,4
Outro	124	9,7
Sem informação	91	7,1
Atuação profissional		
Pesquisa/Ensino superior em Saúde Pública/Saúde Coletiva	516	40,3
Pesquisa/Ensino superior outras áreas	322	25,2
Gestão e serviços de saúde	218	17,0
Outra	137	10,7
Sem informação	87	6,8
Grandes áreas científicas de pesquisa		
Ciências da Saúde	905	70,7
Ciências Sociais Aplicadas	85	6,6
Ciências Humanas	75	5,9
Ciências Biológicas	55	4,3
Ciências Exatas e da Terra	27	2,1
Engenharias	13	1,0
Ciências Agrárias	8	0,6
Linguística, Letras e Artes	2	0,2
Não se aplica	20	1,6
Sem informação	90	7,0

A qualificação acadêmica dos respondentes é elevada, com predomínio de doutores (70,6%) e mestres (17,3%), majoritariamente formados na área da Saúde (72,7%) e com atuação predominante nas Ciências da Saúde (70,7%). Embora em menor proporção, também estiveram representadas áreas como Ciências Sociais Aplicadas, Ciências Humanas e Ciências Biológicas, reforçando o caráter interdisciplinar do periódico. Observa-se, ainda, predominância de vínculos institucionais públicos (55,9%) e de atuação profissional voltada à pesquisa e ao Ensino Superior em Saúde Pública ou Saúde Coletiva (40,3%), confirmando o papel central das instituições públicas na sustentação das atividades editoriais de CSP (Tabela 1). A proporção expressiva de respondentes com formação concluída há menos de dez anos (51,6%) sugere um processo de renovação acadêmica, com potencial para favorecer maior abertura a inovações editoriais.

As percepções sobre a abertura de identidades no processo de revisão evidenciam um cenário de divisão e prudência (Tabela 2). Proporções semelhantes de respondentes manifestaram-se favoráveis e contrários à abertura total de nomes de autores e consultores (23,1% e 24,2%, respectivamente), enquanto uma parcela expressiva preferiu respostas intermediárias, como “caso a caso” ou “não sei” (32,7%; Tabela 2). Além disso, uma fração menor declarou apoiar a abertura apenas da identidade de autores (5,6%) ou apenas de consultores (5,7%), indicando que parte da comunidade distingue assimetrias de risco e exposição entre os diferentes papéis no processo editorial. Esse equilíbrio entre posições antagônicas sugere que a comunidade de CSP reconhece tanto os potenciais ganhos de transparência quanto os riscos de exposição e constrangimento profissional, particularmente em contextos científicos de menor escala ou com relações interpessoais estreitas.

**Tabela 2:** Opinião dos respondentes sobre questões da revisão por pares aberta (n = 1.280).

Questões	n	%
Você é favorável a abrir nomes de autores e/ou consultores?		
Nenhum dos dois	310	24,2
Ambos	296	23,1
Autor	72	5,6
Consultor	73	5,7
Não sei	118	9,2
Caso a caso	301	23,5
Caso a identidade de editores, pareceristas e autores fosse completamente aberta, você se negaria a realizar uma revisão?		
Depende	461	36,0
Não	401	31,3
Não sei	170	13,3
Sim	129	10,1
Em quais situações abaixo se sentiria desconfortável por abrir seu nome no decorrer do processo?		
Autor/Parecerista se conhecem	675	52,7
Conhece o editor	213	16,6
Tema é polêmico	231	18,0
Conflitos de interesse	320	25,0
Questões éticas	316	24,7
Avaliação indicativa de recusa	383	29,9

Quando questionados sobre a disposição em revisar manuscritos sob um sistema totalmente aberto, cerca de um terço afirmou que não se recusaria a fazê-lo (31,3%), mas a resposta predominante foi “depende” (36%; Tabela 2), seguida por proporções menores de incerteza (“não sei”, 13,3%) e recusa explícita (10,1%). Essa predominância de indecisão reforça uma atitude cautelosa e pragmática diante de um modelo ainda pouco difundido nas práticas cotidianas da área. A ausência de rejeição majoritária, entretanto, pode ser interpretada como uma abertura potencial à mudança, desde que acompanhada por diretrizes claras, mediação editorial ativa e medidas de proteção à integridade dos envolvidos.

As situações que geram desconforto confirmam que a principal fonte de apreensão está na possibilidade de conhecimento prévio entre autores e pareceristas (52,7%), seguida por preocupações relacionadas a conflitos de interesse, avaliações que possam resultar em recusa do manuscrito, julgamentos negativos e dilemas éticos. Esses resultados evidenciam que a resistência à revisão aberta não se limita a receios individuais, mas envolve percepções estruturais sobre justiça, imparcialidade e preservação da autonomia crítica no processo editorial. A menor proporção dos que afirmaram não sentir desconforto algum (15,9%) reforça o caráter sensível, relacional e contextual da avaliação por pares aberta.

A análise estratificada por sexo autodeclarado revela diferenças estatisticamente significativas na distribuição das opiniões sobre a abertura de identidades no processo de revisão (teste qui-quadrado, p < 0,05; Figura 1).

**Figura 1:** Opinião em relação à abertura de dados.

Observa-se que as mulheres se concentram proporcionalmente mais nas categorias intermediárias (“caso a caso” e “não sei”), enquanto os homens apresentam maior frequência relativa nas posições extremas, tanto favoráveis quanto contrárias à abertura dos nomes. Esses achados sugerem maior cautela entre as mulheres em relação à adoção de modelos totalmente abertos, especialmente no que se refere à identificação de autores.

## Discussão

Os achados deste estudo confirmam que a revisão por pares atravessa um momento de inflexão histórica, marcado por tensões entre a necessidade de ampliar a transparência, fortalecer a integridade na publicação científica e a sustentabilidade editorial e os limites estruturais do modelo tradicional de avaliação. Conforme amplamente discutido na literatura, a revisão por pares, concebida no século XVIII como prática restrita a elites científicas, tornou-se progressivamente inadequada diante da expansão global da ciência, do crescimento exponencial das submissões e da intensificação das demandas sobre um número cada vez mais restrito de revisores experientes ^15^ ^,^ ^16^.

Os resultados mostram que essas contradições são claramente percebidas pela comunidade científica vinculada à CSP. A elevada qualificação acadêmica dos respondentes, majoritariamente doutores, com inserção em instituições públicas e atuação consolidada na Saúde Coletiva, confere robustez às opiniões expressas. Trata-se de um grupo envolvido nas dinâmicas de produção, avaliação e circulação do conhecimento científico, o que torna particularmente relevante a predominância de posições intermediárias e cautelosas frente à OPR.

A ausência de polarização entre posições claramente favoráveis ou contrárias à abertura das identidades de autores e pareceristas reforça achados prévios da literatura nacional. Estudos com editores de periódicos científicos brasileiros indicam que a aceitação da OPR tende a ser condicionada ao contexto editorial, à cultura disciplinar e às características das comunidades científicas envolvidas, sendo recorrente a defesa de modelos graduais e diversos de implementação ^17^ ^,^ ^18^. A expressiva proporção de respostas do tipo “caso a caso” ou “não sei” observada neste estudo sugere que a comunidade da CSP compartilha dessa perspectiva pragmática e reflexiva.

A relutância parcial em revisar manuscritos em um sistema totalmente aberto também dialoga com a literatura internacional e nacional. Embora apenas uma minoria declare que se recusaria explicitamente a revisar sob OPR, a predominância da resposta “depende” indica que a adesão ao modelo não ocorrerá de forma automática. Esse resultado se alinha às críticas à chamada “esteira da revisão por pares”, caracterizada pela sobrecarga de revisores, pela baixa valorização acadêmica dessa atividade e pela ausência de incentivos institucionais adequados ^15^. Nesse cenário, a OPR, se implementada sem salvaguardas, pode agravar a escassez de revisores, sobretudo em áreas com comunidades científicas relativamente pequenas e fortemente interconectadas.

As situações de desconforto relatadas pelos respondentes aprofundam essa análise. A principal fonte de apreensão - o conhecimento prévio entre autores e pareceristas - evidencia que o debate sobre OPR não se restringe à transparência formal, mas envolve dimensões éticas, relacionais e estruturais da ciência. Estudos destacam que, embora a abertura possa aumentar a responsabilização e a qualidade argumentativa dos pareceres, ela também pode inibir críticas mais contundentes, especialmente quando há assimetrias de poder, relações hierárquicas ou vínculos institucionais prévios ^7^ ^,^ ^19^.

As diferenças por sexo autodeclarado revelam que o viés de gênero persiste mesmo em revisões por pares abertas ^20^ ^,^ ^21^. A predominância feminina em posições intermediárias reflete cautela diante de assimetrias estruturais e riscos reputacionais ^22^, já que pesquisadoras enfrentam maior exposição a julgamentos interpessoais e relações de poder desiguais ^23^.

No contexto brasileiro, essas preocupações são particularmente relevantes. Pesquisas com editores de periódicos indexados no Directory of Open Access Journals (DOAJ - Diretório de Revistas de Acesso Aberto; https://doaj.org/) indicam que a OPR tende a ser percebida como eticamente desejável, mas operacionalmente complexa, exigindo mediação editorial cuidadosa, definição clara de papéis e fortalecimento da dimensão dialógica do processo avaliativo ^17^ ^,^ ^24^. Experiências concretas relatadas por periódicos nacionais, como a revista *Ensaio Pesquisa em Educação em Ciências*, demonstram que a OPR pode ser bem-sucedida quando acompanhada de comunicação transparente, capacitação dos avaliadores e reconhecimento explícito do trabalho de revisão ^25^.

No plano internacional, a literatura reforça que não existe um modelo único de OPR. Diferentes arranjos - como a publicação dos pareceres sem identificação obrigatória dos revisores, a abertura opcional de identidades ou a divulgação dos diálogos editoriais após a decisão final - têm sido adotados com resultados variados ^16^ ^,^ ^19^. Esses modelos intermediários são frequentemente apontados como alternativas viáveis para ampliar a transparência sem comprometer a autonomia crítica dos pareceristas, especialmente em áreas sensíveis ou altamente relacionais.

No contexto latino-americano, e particularmente no âmbito dos periódicos de acesso aberto diamante como CSP, a discussão sobre OPR adquire contornos específicos. Diferentemente dos grandes periódicos comerciais, CSP opera sob princípios de ciência como bem público, sustentado por instituições públicas e comprometido com valores de equidade, inclusão e responsabilidade social. Nesse sentido, a OPR deve ser compreendida não apenas como inovação técnica, mas como prática ética e política, alinhada aos princípios da Ciência Aberta e às recomendações de iniciativas regionais e globais de acesso aberto não comercial ^13^.

Por fim, os resultados deste estudo indicam que a eventual adoção da OPR em CSP deve ser precedida de amplo diálogo com a comunidade. Ações formativas contínuas, definição clara de critérios editoriais e mecanismos de proteção aos revisores, especialmente pesquisadores em início de carreira e aqueles pertencentes a grupos historicamente sub-representados, devem ser considerados. A abertura, para cumprir sua promessa de maior integridade e confiança pública na ciência, precisa ser acompanhada de responsabilidade institucional, reconhecimento do trabalho editorial e valorização efetiva da revisão por pares como atividade central da prática científica.

Como limites do estudo, destaca-se a participação voluntária, que pode ter gerado viés na caracterização da população, o que restringe a generalização dos resultados. Além disso, o delineamento transversal, capta as percepções em um momento específico do debate sobre a OPR, tema esse que está em rápida evolução, indicando a necessidade de estudos futuros que permitam acompanhar o desdobramento da questão na comunidade acadêmica no Brasil.

O artigo contribui para o debate contemporâneo sobre o tema ao demonstrar que o futuro da revisão por pares não reside na simples substituição de um modelo por outro, mas na construção coletiva de arranjos mais transparentes, justos e sustentáveis, sensíveis às especificidades da Saúde Coletiva, além de coerentes com os princípios da ciência aberta.

## Data Availability

Os bancos de dados utilizados no estudo, incluindo os códigos de extração, análises e resultados estão disponíveis em repositório: https://github.com/haleyjeppson/ggmosaic..
